# Maneuvers on PCNA Rings during DNA Replication and Repair

**DOI:** 10.3390/genes9080416

**Published:** 2018-08-17

**Authors:** Dea Slade

**Affiliations:** Department of Biochemistry, Max F. Perutz Laboratories, University of Vienna, Vienna Biocenter (VBC), 1030 Vienna, Austria; dea.slade@univie.ac.at

**Keywords:** proliferating cell nuclear antigen, DNA replication, DNA repair, post-translational protein modifications

## Abstract

DNA replication and repair are essential cellular processes that ensure genome duplication and safeguard the genome from deleterious mutations. Both processes utilize an abundance of enzymatic functions that need to be tightly regulated to ensure dynamic exchange of DNA replication and repair factors. Proliferating cell nuclear antigen (PCNA) is the major coordinator of faithful and processive replication and DNA repair at replication forks. Post-translational modifications of PCNA, ubiquitination and acetylation in particular, regulate the dynamics of PCNA-protein interactions. Proliferating cell nuclear antigen (PCNA) monoubiquitination elicits ‘polymerase switching’, whereby stalled replicative polymerase is replaced with a specialized polymerase, while PCNA acetylation may reduce the processivity of replicative polymerases to promote homologous recombination-dependent repair. While regulatory functions of PCNA ubiquitination and acetylation have been well established, the regulation of PCNA-binding proteins remains underexplored. Considering the vast number of PCNA-binding proteins, many of which have similar PCNA binding affinities, the question arises as to the regulation of the strength and sequence of their binding to PCNA. Here I provide an overview of post-translational modifications on both PCNA and PCNA-interacting proteins and discuss their relevance for the regulation of the dynamic processes of DNA replication and repair.

## 1. Proliferating Cell Nuclear Antigen Serves as the Master Coordinator of DNA Replication and DNA Repair

DNA replication is an essential cellular process that enables the duplication of genomic material necessary for cell division. Equally essential is DNA repair, which maintains genomic integrity by repairing damaged DNA. These processes entail dynamic binding of DNA replication factors that ensure processive and faithful replication, and DNA repair factors that accurately and efficiently repair DNA. Dynamic protein interactions often require a master coordinator responsible for their timely and precise recruitment; proliferating cell nuclear antigen (PCNA) plays such a scaffold role in DNA replication and a subset of DNA repair pathways (translesion synthesis, homologous recombination, mismatch repair, base, and nucleotide excision repair).

**Figure 1:**
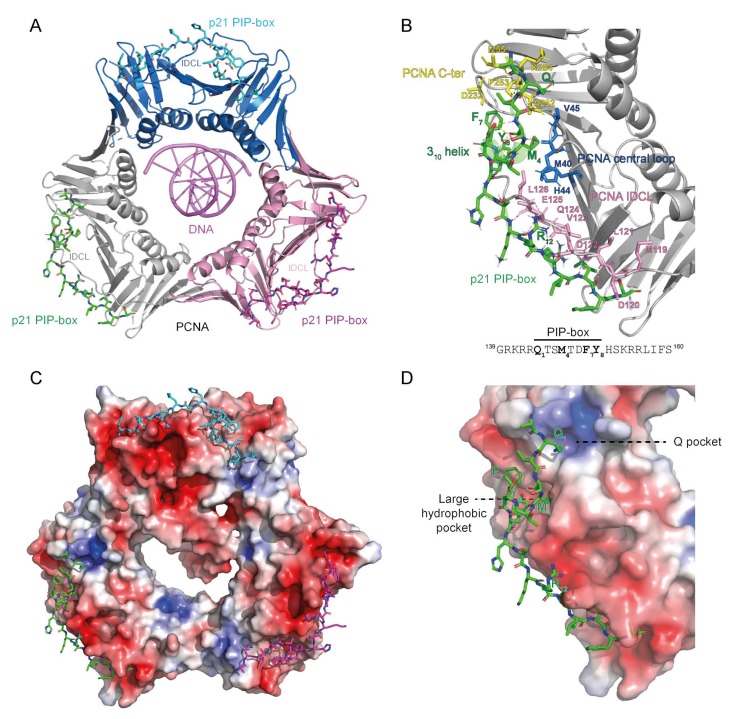
The structure of the proliferating cell nuclear antigen (PCNA) ring bound to DNA and the PIP-box of the CDK inhibitor p21. (**A**) Cartoon presentation of PCNA homotrimer bound to 10 bp dsDNA and p21 PIP-box peptide bound to the interdomain connector loop (IDCL) of each PCNA monomer. The image was obtained by overlaying PCNA-DNA co-structure (6GIS) [5] with PCNA-p21 PIP co-structure (1AXC) [31]. Three PCNA monomers are represented with different colors. (**B**) Interaction interface between PCNA and PIP-box shown for one PCNA monomer bound by one p21 PIP-box peptide. IDCL (pink), the central loop region (blue) and the C-terminal region (yellow) of PCNA anchor the PIP-box peptide through hydrophobic and electrostatic interactions. The sequence of the p21 PIP-box peptide is shown with the four critical residues indicated in bold. (**C,D**) Electron density distribution of PCNA from (**A**,**B**). The color-coded electrostatic surface potential of PCNA was drawn using the Adaptive Poisson-Boltzmann Solver package. The electrostatic potential ranges from −5 (red) to +5 (blue) kT/e. The images were generated using PyMOL [32].

Proliferating cell nuclear antigen (PCNA) is a ring-shaped homotrimer that encircles and slides along DNA, hence the name DNA sliding clamp [1,2,3,4] (Figure 1). Basic residues at the inner surface of the PCNA ring establish polar interactions with consecutive DNA phosphates by forming a right-hand spiral that matches the pitch of B-DNA (right-handed double helix with ~10 bp per turn) [5]. The outer surface of the PCNA ring is implicated in the recruitment of various DNA replication and repair factors. Among the many proteins interacting with PCNA are DNA polymerases, helicases, exonucleases, ligases, cell cycle regulators, acetyltransferases, chromatin remodelers, and histone chaperones [1,6].

**Figure 2:**
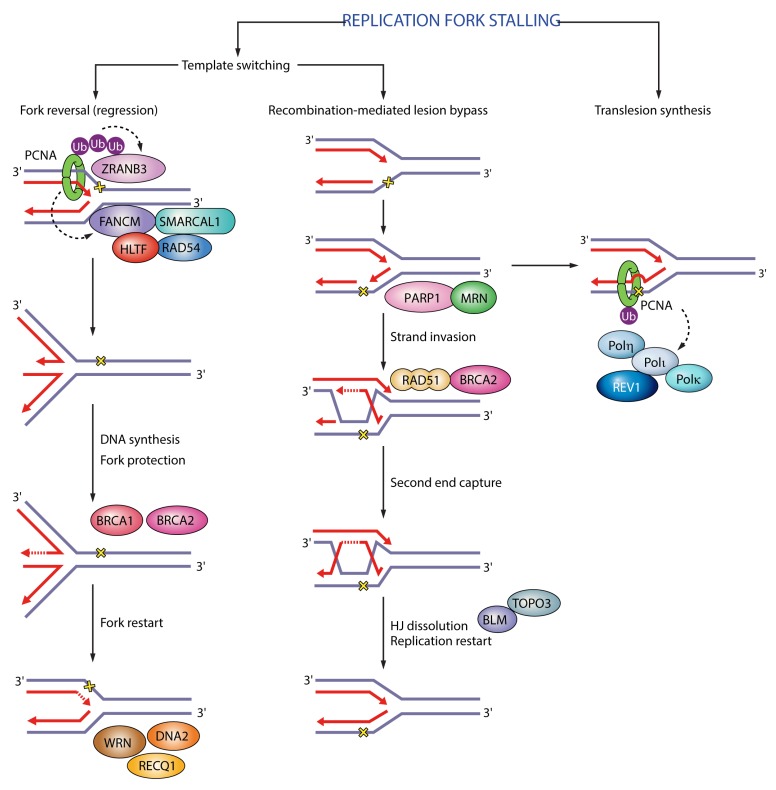
Functions of PCNA in replication fork stalling. In fork reversal polyubiquitinated PCNA recruits ZRANB3 translocase/structure-specific endonuclease and FANCM. In translesion synthesis monoubiquitinated PCNA recruits translesion synthesis (TLS) polymerases η, κ, ι and REV1 to enable bypass of DNA lesions. Black dashed arrows indicate PCNA targets in different pathways. Ub (ubiquitin); SMARCAL1 (SWI/SNF-related matrix-associated actin-dependent regulator of chromatin subfamily A-like protein 1); HLTF (helicase-like transcription factor); RAD54 (DNA repair and recombination protein RAD54); BRCA1 (breast cancer type 1 susceptibility protein); BRCA2 (breast cancer type 2 susceptibility protein); WRN (Werner syndrome ATP-dependent helicase); RECQ1 (ATP-dependent DNA helicase Q1); DNA2 (DNA replication ATP-dependent helicase/nuclease DNA2); PARP1 (poly(ADP-ribose) polymerase 1); MRN (Mre11-Rad50-Nbs1); RAD51 (DNA repair protein RAD51); BLM (Bloom syndrome protein); TOPO3 (DNA topoisomerase 3).

**Figure 3:**
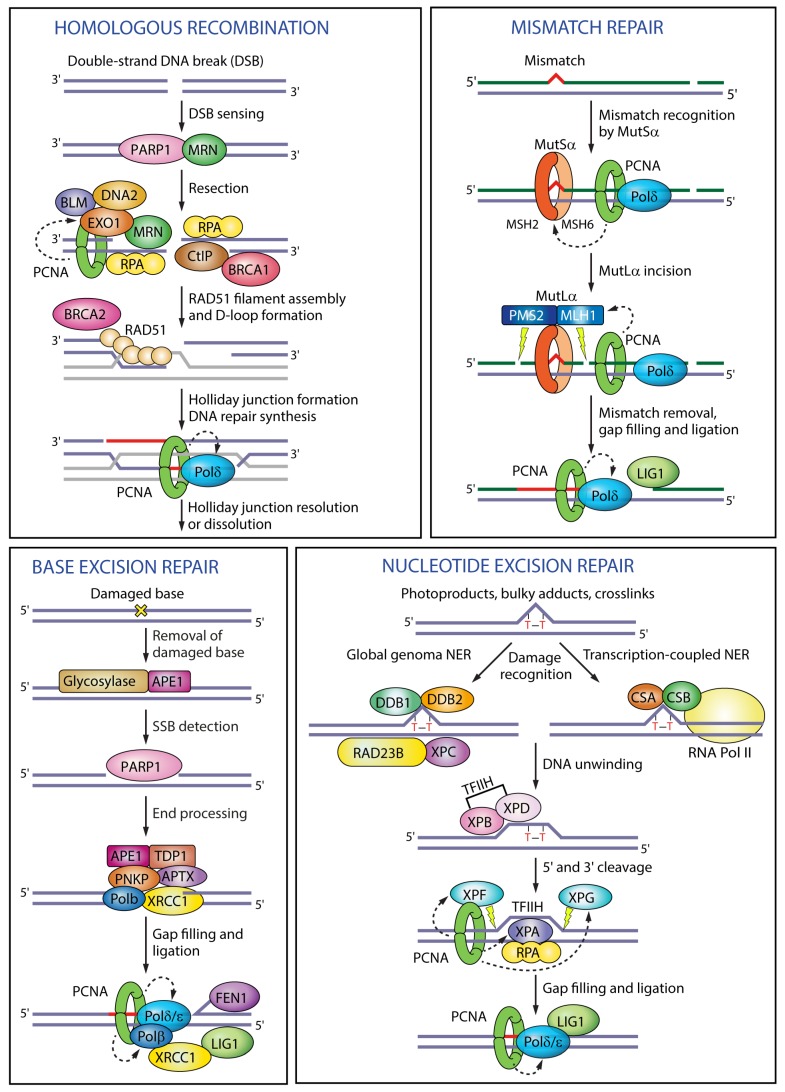
Functions of PCNA in different DNA repair pathways**.** In homologous recombination PCNA enhances the processivity of the exonuclease EXO1 during end resection and of Pol δ during DNA repair synthesis. In mismatch repair, PCNA interacts with MutSα to recognize the mismatch, activates the endonuclease activity of MutLα to excise the mismatch, and recruits polymerase δ for DNA repair synthesis. In base excision repair, PCNA recruits polymerases β, δ or ε to displace the damaged base into a flap intermediate. In nucleotide excision repair PCNA interacts with the scaffold protein XPA, activates the endonuclease XPF, targets XPG for degradation and recruits polymerase δ to fill in the gap. Black dashed arrows indicate PCNA targets in different pathways. RPA (replication protein A); CtIP (CtBP-interacting protein); MSH2 (MutS homologue 2); MSH6 (MutS homologue 6); MLH1 (MutL homologue 1); PMS2 (PMS1 homologue 2); APE1 (apurinic/apyrimidinic endonuclease 1); TDP1 (tyrosyl-DNA phosphodiesterase 1); PNKP (polynucleotide kinase 3′-phosphatase); APTX (aprataxin); XRCC1 (X-ray repair cross-complementing protein 1); DDB1 (DNA damage-binding protein 1); DDB2 (DNA damage-binding protein 2); RAD23B (RAD23 homologue B); XPC (Xeroderma pigmentosum complementation group C); CSA (Cockayne syndrome group A); CSB (Cockayne syndrome group B); TFIIH (transcription factor II H); XPB (Xeroderma pigmentosum complementation group B); XPD (Xeroderma pigmentosum complementation group D).

In DNA replication, PCNA tethers DNA polymerases ε and δ and increases their processivity by sliding along the double-stranded DNA helix [3]. PCNA is particularly important for lagging strand synthesis where it interacts with DNA polymerase δ, FEN1 (flap endonuclease 1) and LIG1 (DNA ligase I) to synthesize, process and join Okazaki fragments [3]. In translesion synthesis, PCNA recruits Y-family translesion synthesis (TLS) polymerases η, κ, ι and REV1 (DNA repair protein REV1) to enable bypass of DNA lesions that block replication fork progression, serving both a scaffold function and an active function in stimulating catalytic activity [7,8] (Figure 2). PCNA protects arrested forks from collapse and promotes replication traverse of DNA interstrand crosslinks (ICL) by recruiting FAN1 (Fanconi-associated nuclease 1) and FANCM (Fanconi anemia group M protein) as an activator of the Fanconi anemia pathway [9,10], promotes ICL repair by recruiting the nuclease SNM1A (DNA cross-link repair 1A protein) [11], and facilitates replication fork reversal required for fork restart by recruiting the translocase ZRANB3 (zinc finger RANBP2-type containing 3) [12,13] (Figure 2). In homologous recombination, PCNA enhances the processivity of Pol δ and Pol κ during DNA repair synthesis [14] or EXO1 (exonuclease 1) during resection [15] (Figure 3). In base excision repair (BER), PCNA recruits polymerases β, δ or ε to displace the damaged base into a flap intermediate [16]; in nucleotide excision repair (NER) PCNA interacts with the scaffold protein XPA (Xeroderma pigmentosum complementation group A), activates the endonuclease XPF (Xeroderma pigmentosum complementation group F), targets XPG (Xeroderma pigmentosum complementation group G) for degradation and recruits polymerase δ to fill in the single-stranded gap [1]; in mismatch repair, PCNA interacts with the MutSα complex to recognize the mismatch, activates the endonuclease activity of MutLα to excise the mismatch, and recruits polymerase δ for DNA repair synthesis [3] (Figure 3).

Given the vast number of PCNA-binding proteins, the question that naturally arises is: ‘How are the strength and the temporal sequence of their binding orchestrated?’

## 2. Protein Interaction Interfaces on Proliferating Cell Nuclear Antigen

Each PCNA monomer contains two similar domains connected by the interdomain connector loop (IDCL). IDCL serves as the common PCNA-protein interaction interface at the front face of the PCNA ring pointing in the direction of DNA synthesis (Figure 1A,B) [1,2,3].

The most prevalent IDCL-binding module is the so-called PCNA-interacting protein motif (PIP-box), defined by the consensus sequence Q_1_-x_2_-x_3_-h_4_-x_5_-x_6_-a_7_-a_8_, where ‘h’ and ‘a’ represent hydrophobic (ILMV) and aromatic (FYH) amino acids, respectively [17] (Figure 1B and Figure 2). AlkB homologue 2 PCNA Interacting Motif (APIM) shares the binding interface, topology and common micromolar affinity with the PIP-box and is found in many DNA repair proteins [18,19].

**Figure 4:**
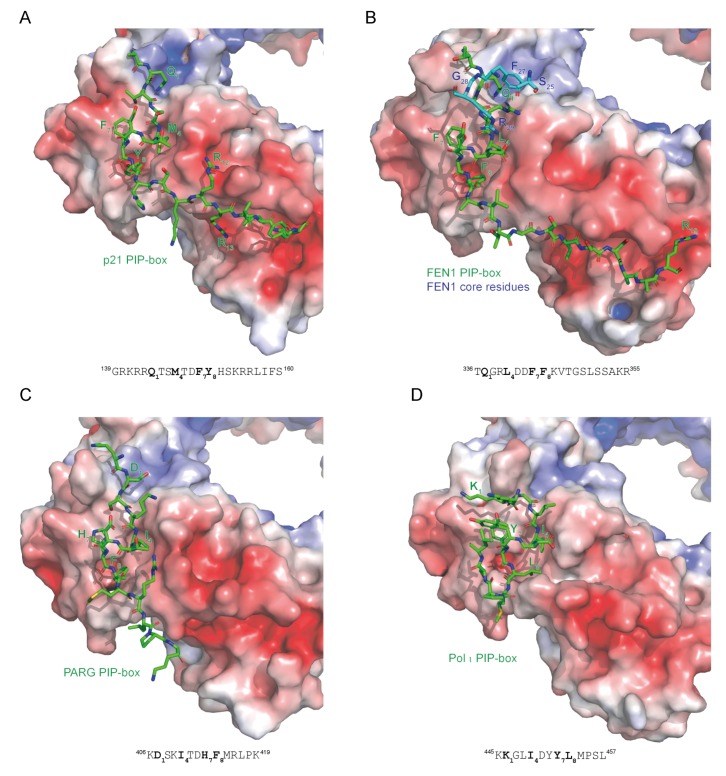
Different binding modes of PIP-box motifs to PCNA. Electrostatic surface potential of the interaction interface on PCNA and stick representation of PIP-box peptides in green are shown for (**A**) p21 (1AXC) [31], (**B**) FEN1 [38], (**C**) PARG [27], and (**D**) DNA polymerase τ (2ZVM) [21]. The sequences of PIP-box peptides are shown with the four critical residues indicated in bold. The co-structure of PCNA and full-length FEN1 reveals additional binding sites within the FEN1 core, indicated in blue. PIP-boxes from p21 and FEN1 have a canonical sequence and a canonical mode of binding; PARG has a non-canonical sequence but a canonical mode of binding; Pol τ has a non-canonical sequence and a non-canonical mode of binding. The color-coded electrostatic surface potential of PCNA was drawn using the Adaptive Poisson-Boltzmann Solver package. The electrostatic potential ranges from −5 (red) to +5 (blue) kT/e. The images were generated using PyMOL [32].

PCNA-interacting protein motifs can be classified into ‘canonical‘ and ‘non-canonical’ based on their sequence complying to or diverging from the consensus sequence. Canonical PIP-boxes have Q as the first residue and F or Y aromatic amino acids at the 7th and 8th position. The canonical Q_1_ is docked into a ‘Q pocket’, while residues h_4_-a_8_ form a 3_10_ helix where h_4_, a_7_ and a_8_ are anchored in a large hydrophobic pocket formed by IDCL, the central loop region and the C-terminal region (Figure 1B,D). Non-canonical PIP-boxes may lack Q_1_ (e.g., Pol η, κ, τ, poly (ADP-ribose) glycohydrolase (PARG), RNASEH2B) and/or one or both aromatic amino acids a_7_/a_8_ (e.g., Pol τ, FANCM, the E3 ubiquitin-protein ligase TRAIP, the ATP-dependent DNA helicase Srs2) (Table 1 and Figure 4). This may result in a different mode of binding as in the case of Pol τ and Srs2 [20,21] (Figure 4D). However, PIP-boxes with non-canonical sequence properties may exhibit a canonical mode of binding (example given by PARG in Figure 4C); classification according to the mode of binding rather than the sequence properties may thus be more appropriate.

Sequence variations among PIP-boxes modulate the degree of hydrophobic packing of PIP-boxes into the hydrophobic PCNA cleft [2]. For example, a TD motif at positions 5 and 6 in the PIP-boxes of the CDK inhibitor p21 [22], the replication licensing factor Cdt1 [23], the methyltransferase SET8 [24], thymine DNA glycosylase [25,26], and PARG [27] confers high binding affinity (Table 1). Due to its much higher PCNA-binding affinity, p21 can displace replicative polymerases from DNA [22]. Interestingly, all three TD-motif containing proteins contain a degron characterized by a positively charged residue (K or R) at position 4 downstream of the PIP-box, called a ‘PIP degron’ [23]. While in the case of p21 and Cdt1 this degron recruits the ubiquitin ligase CRL4-Cdt2, which mediates S-phase and DNA damage-dependent degradation of these proteins [23], the PARG degron is not functional (Slade, unpublished [28]). Mutation of the first residue in the Pol η or PARG non-canonical PIP-box to the canonical Q, as well as mutation of H in the PARG PIP-box to the canonical F or Y increase the binding affinity, confirming that sequence variations of PIP-boxes modify their PCNA binding strength [21,27].

However, increased affinity or sustained binding to PCNA may have severe phenotypic consequences, as shown by reduced proliferation due to the overexpression of the p21 PIP-box [29]. Alternatively, mutation of IDCL residues in PCNA may also result in increased affinity for various PCNA-binding proteins, leading to increased sensitivity to DNA-damaging agents such as hydroxyurea (HU) and methyl methanesulfonate (MMS) [30]. Overall, the regulation of the strength of binding to PCNA is critical for normal cellular functions.

The majority of characterized PIP-box peptides exhibit micromolar binding affinity towards PCNA (K_d_ = 1–60 μM), p21 being the notable exception with K_d_ = 80–560 nM [19,22,33] (Table 1). Regulation of the strength and duration of interactions with PCNA can thus be only partially facilitated by differential binding affinities, which necessitates the existence of additional levels of regulation.

For some proteins, interaction with PCNA extends outside of the commonly employed motifs. C-terminal flanking residues of p21 and FEN1 PIP-box form an extensive binding interface with IDCL residues [38,41,42] (Figure 4A,B). FEN1 core domain makes additional contacts with PCNA (blue residues in Figure 4B), resulting in the binding affinity of full-length FEN1 being 1000-fold higher (K_d_ = 60 nM) compared to just the PIP-box peptide [38,43]. PCNA-associated factor (PAF15) interaction with PCNA also includes residues outside of the PIP-box, although the full-length protein shows only a 5-fold increase in affinity compared to the PIP-box peptide (K_d_ = 1.1 μM for full-length and 5.6 μM for the extended PIP-box peptide) [34]. Binding between PCNA and PARG is likely also modulated by residues outside of the PIP-box considering the partial discrepancy between in vitro binding data using short PIP-box peptide and in vivo data with the full-length protein [27].

**Figure 5:**
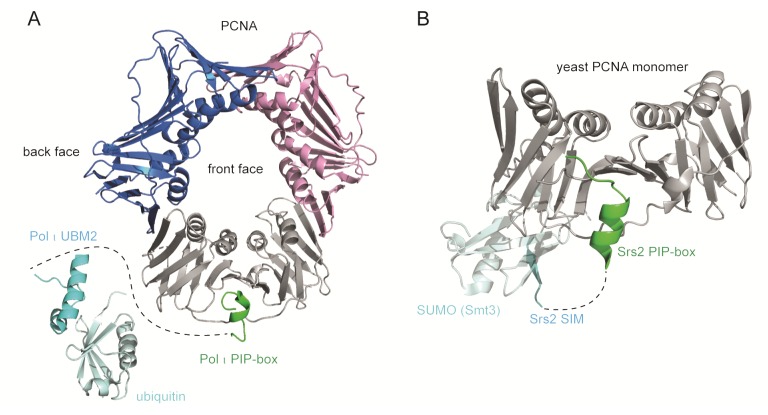
Multivalent binding modules of PCNA-interacting proteins. (**A**) TLS polymerases bind PCNA IDCL through a PIP-box motif and monoubiquitinated PCNA through ubiquitin-binding domains (UBD). Overlay of monoubiquitinated PCNA (3TBL) [46], Pol τ PIP-PCNA co-structure (2ZVM) [21], and Pol τ UBM2-ubiquitin co-structure (2KHW) [62]. Pol τ PIP-box binds the IDCL at the front face of PCNA, while Pol τ UBM2 binds ubiquitin positioned radially towards the back face of PCNA. A dashed line represents the region between Pol τ PIP-box (445–457aa) and UBM2 (701–732aa). The 59Å distance between the last residue of the PIP-box and the first residue of UBM2 allows a flexible conformation of the intervening region. (**B**) Yeast Srs2 binds PCNA IDCL through a PIP-box motif (1148–1161aa) and SUMOylated PCNA on the back face of the PCNA ring through a SUMO-interacting motif (SIM) (1168–1174aa) (3V62) [20]. However, the 36Å distance between the last residue of the PIP-box and the first residue of SIM is too large to be filled by the six intervening residues, rendering a multivalent binding mode implausible according to this structure. The images were generated using PyMOL [32].

Some PCNA-binding proteins contain additional PCNA-binding modules such as ubiquitin-binding domains (UBD) and SUMO-interacting motif (SIM) that confer higher affinity for ubiquitinated or SUMOylated PCNA respectively [7,20,44,45] (Figure 5). PCNA is ubiquitinated on K164 in response to replication stress-inducing agents (see the following chapter). Ubiquitin bound to PCNA projects towards the back face of PCNA, away from the PIP binding site, and may assume various conformations to support dynamic recruitment and exchange of ubiquitin-binding proteins [46,47,48,49]. PCNA is SUMOylated on K164 during S-phase whereby SUMO adopts extended flexible conformation and projects towards the back face of PCNA as in the case of ubiquitin [50]. Ubiquitin and SUMO thus generate new interaction interfaces on PCNA. The co-occurrence of multiple PCNA-binding motifs in the same protein may promote stable binding, enable appropriate positioning on the DNA template, impede other proteins from interacting with PCNA, or enable interaction with more than one PCNA ring.

Ubiquitin-binding motif (UBM) and ubiquitin-binding zinc finger (UBZ) bind ubiquitinated PCNA and are found in TLS polymerases [45,51,52], the SNM1A nuclease [11], the ZRANB3 translocase/structure-specific endonuclease [12,19,53], the FAN1 nuclease [9], and the ATPase WRNIP1 [54]. Unlike other TLS polymerases, REV1 is targeted to PCNA through the BRCT domain instead of a PIP-box that nonetheless occupies the same binding site on PCNA as the PIP-box [55,56]. Pol τ and REV1 have two functional UBMs, while Pol η, Pol κ, SNM1A, FAN1, and WRNIP1 have a UBZ [9,11,45,54,57]. Among TLS polymerases, Pol η shows the highest affinity for monoubiquitinated PCNA [58]. Of all known UBD-containing PCNA binders, only WRNIP1 displays monovalent interaction with PCNA through its UBZ [54] and thus acts as a mediator between PCNA and the ATM cofactor ATMIN [59]. ZRANB3 harbors three PCNA-binding domains; PIP and APIM motifs bind the IDCL region of PCNA whereas a UBD called NPL4 zinc finger (NZF) binds K63-linked polyubiquitin chains [12,19]. Both the PIP-box/APIM/BRCT and UBDs mediate targeting of all the above proteins to PCNA in unperturbed S-phase cells and in response to DNA damage, with the exception of REV1 where the BRCT domain is sufficient for constitutive recruitment of REV1 to replication foci [9,11,12,45,51,52,53,54,55,57,60,61].

**Figure 6:**
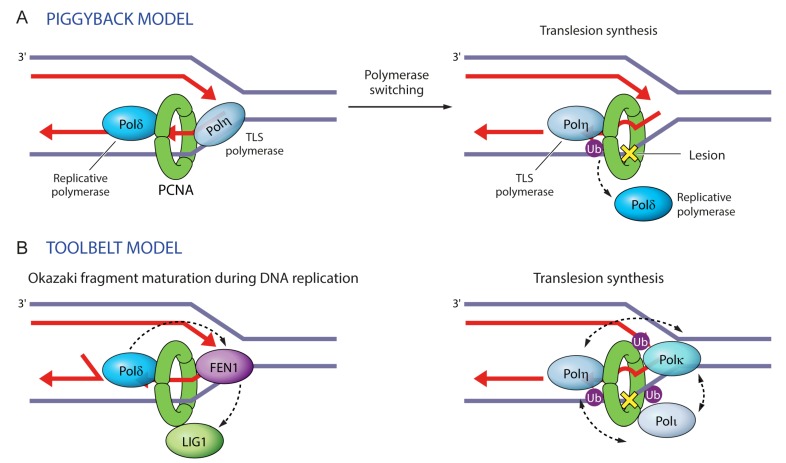
Two models illustrating protein maneuvers on PCNA rings. (**A**) In the ‘piggyback model’ a replicative polymerase binds to the front face of PCNA in the direction of DNA replication, whereas a TLS polymerase rides piggyback. Upon DNA damage, PCNA monoubiquitination instigates polymerase switching, whereby the replicative polymerase dissociates and is replaced by a TLS polymerase, which bypasses the lesion. (**B**) In the ‘toolbelt model’, PCNA trimers can accommodate simultaneously up to three proteins. This can facilitate their coordinate exchange, as in the case of Pol β, FEN1, and LIG1 during Okazaki fragment maturation. This can also facilitate the selection of the appropriate TLS polymerase (Pol η, Pol κ or Pol τ) during translesion synthesis.

Given the multivalent binding sites on PCNA and its trimeric structure, the questions arise as to the relative importance of different binding sites and the co-recruitment or competition among PCNA-binding proteins. TLS polymerases as the best characterized multivalent PCNA-binding proteins may provide answers to these questions. In vivo experiments conducted with different TLS polymerases demonstrated higher affinity towards ubiquitinated PCNA [21,45,51,52,57,58,60,63], whereas in vitro fluorescence resonance energy transfer (FRET) experiments with purified proteins showed equal affinity of the TLS polymerase Pol η to ubiquitinated or non-ubiquitinated PCNA [64]. This may suggest that preferential binding of TLS polymerases to ubiquitinated PCNA is an indirect effect of ubiquitin moieties, which may alter the chromatin structure or block the binding of nucleosome assembly factors to facilitate access to DNA lesions [64]. Regarding their co-occupancy with other PCNA-binding partners, TLS polymerases may ride piggyback on the PCNA ring until the replicative polymerase Pol δ encounters a lesion and is displaced by the catalytic core of a TLS polymerase [48] (Figure 6A). Alternatively, TLS polymerases may be recruited specifically upon DNA damage-induced PCNA monoubiquitination [63]. Given that TLS polymerases can bind non-ubiquitinated PCNA via their PIP-box, albeit with lower affinity in vivo [45,51], and that PCNA is monoubiquitinated at lower levels in undamaged cells [52,60], it seems likely that TLS polymerases travel with the replicative polymerases. Indeed, Pol η was recently shown to travel with replication forks [65]. Upon DNA damage, Pol η recruits the ubiquitin ligase Rad18 to PCNA and facilitates DNA damage-induced monoubiquitination of PCNA by Rad6-Rad18 independently of its catalytic activity [66]. Increased PCNA monoubiquitination increases chromatin binding and replication foci formation of TLS polymerases, resulting in increased retention at replication forks [52,58,60,63,66,67]. The residence time of TLS polymerases being <1 s may promote a rapid exchange between different TLS polymerases until the encounter of a cognate DNA lesion stabilizes the relevant polymerase [64,67]. In addition, degradation or dissociation of different proteins may facilitate TLS. For example, Cdt1 degradation by CRL4-Cdt2 facilitates Pol η and Pol κ foci formation [68]. PAF15, which inhibits lesion bypass presumably by restricting the effective diameter of the PCNA channel, is degraded to allow TLS [4,69]. Pol δ holoenzyme is unstable and dissociates rapidly upon DNA lesion encounter [70].

Complementation assays with Pol η in Xeroderma pigmentosum variant (XP-V) cells showed that Pol η bearing mutations in either the PIP-box or the UBZ can partially complement ultraviolet (UV) sensitivity of XP-V cells, whereas their combined mutation cannot rescue the XP-V phenotype [61,71]. Similarly, concomitant mutation of BRCT and UBM in REV1 failed to complement UV sensitivity of REV1-deleted DT40 cells [57]. Different PCNA-binding modules thus seem to work cooperatively to stabilize interaction with PCNA in vivo.

With regard to the co-recruitment or competition among PCNA-binding proteins, the trimeric ring of PCNA where all monomers can be ubiquitinated may function as a ‘tool belt’ by allowing the binding of different TLS polymerases simultaneously [72,73] (Figure 6B). Simultaneous rather than sequential or competitive binding of TLS polymerases to PCNA would allow them to act together to bypass DNA lesions and would facilitate the selection of the appropriate polymerase and polymerase switching events [72,73]. The selection of the appropriate polymerase is most likely determined by the kinetics of nucleotide incorporation, as described by the ‘kinetic selection’ model [74]. While PCNA monoubiquitination does not interfere with the binding of Pol δ or RFC (replication factor C) and thus allows for the co-existence of TLS and replicative polymerases on the PCNA clamp, it hinders the binding of FEN1 [46], consistent with the existence of additional FEN1 contacts with PCNA that extend outside of its PIP-box [38,43].

## 3. Regulation of Proliferating Cell Nuclear Antigen-Mediated Interactions by Post-Translational Modifications

Transient protein-protein interactions appear to be crucial for the dynamics of DNA replication and repair pathways and the same applies to PCNA-mediated interactions. Prolonged PCNA binding may, on the one hand, hinder the recruitment of other crucial DNA replication or repair factors, and, on the other, result in prolonged enzyme activity with deleterious consequences for DNA replication or repair. Post-translational modifications (PTMs) of both PCNA and its binding partners may explain how PCNA-bound proteins cycle between bound and unbound states during the dynamic process of DNA replication and repair, especially when the downstream or stress-induced PCNA-binding factor cannot outcompete the bound factor due to equal or lower binding affinity.

Post-translational modifications (PTMs) may regulate protein activity, stability, cellular localization, protein-protein as well as protein-nucleic acid interactions. Quantitative proteomics has shown that many proteins involved in DNA replication and DNA damage response are regulated by phosphorylation, acetylation, ubiquitination, SUMOylation, and ADP-ribosylation, in response to various types of DNA damage such as ionizing radiation, UV or hydrogen peroxide [75,76,77,78]. Lysine acetylation, ubiquitination, SUMOylation and ADP-ribosylation exert their effect by eliminating the positive charge from the lysine side chain, by steric hindrance, or by recruitment of proteins with designated binding modules [79].

## 4. Post-Translational Modifications of Proliferating Cell Nuclear Antigen

**Figure 7:**
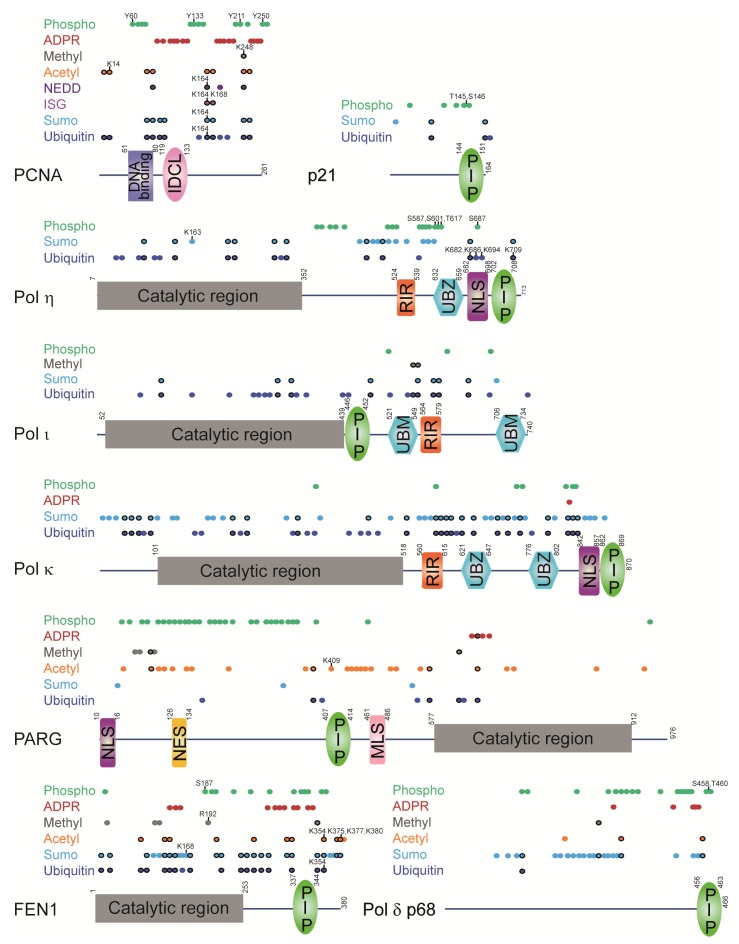
Regulatory regions in PCNA-interacting proteins have PCNA-interacting modules and are modified by different PTMs. PCNA-interacting modules: PIP (PCNA-interacting protein motif); UBZ (ubiquitin-binding zinc finger motif); UBM (ubiquitin-binding motif); RIR (Rev1-interacting region motif). Other modules: IDCL (interdomain connector loop); NLS (nuclear localization signal); NES (nuclear export signal); MLS (mitochondrial localization signal). Colored dots represent modified residues (precise location is given in Supplementary Table S1). Black-circled dots denote overlapping modifications. Dots labelled with numbers indicate residues that were functionally characterized. PARG has an additional PIP-box at the N-terminus, which does not bind PCNA in vitro but is important for PARG localization within replication foci [27,141]. Pol η has two more internal PIP-boxes, which do now show binding to PCNA in vitro, but are important for PCNA monoubiquitination and stimulation of Pol η polymerase activity by PCNA [58]. PTMs were extracted from high-throughput mass spectrometry studies or low-throughput functional studies [69,71,76,78,80,81,82,83,84,85,86,87,88,89,90,91,92,93,94,95,96,97,98,99,100,101,102,103,104,105,106,107,108,131,132,133,134,135,136,137,138,139,142,143,144,145,146,147,148,149,150,151,152,153,154,155,156,157,158,159,160,161,162,163,164].

Mass spectrometry analyses revealed that of 16 lysines in PCNA, 13 can be ubiquitinated, of which eight can be SUMOylated or acetylated, four can be NEDDylated, and three can be ISGylated [69,76,80,81,82,83,84,85,86,87,88,89,90,91,92,93,94,95,96,97,98,99,100,101,102,103,104,105,106,107] (Figure 7 and Supplementary Table S1). One of the residues, K248, can apparently bear five different modifications: ubiquitination, SUMOylation, NEDDylation, acetylation or methylation [108]. As all these different modifications on the same residue are mutually exclusive, dynamic switching between them may regulate PCNA interactions with other proteins, may affect PCNA levels or chromatin binding [1]. Importantly, ubiquitination is crucial for regulating PCNA functions in DNA damage response, whereby two PCNA residues experience a pronounced increase in ubiquitination following UV damage: K117 and K164 [76,109].

**Figure 8:**
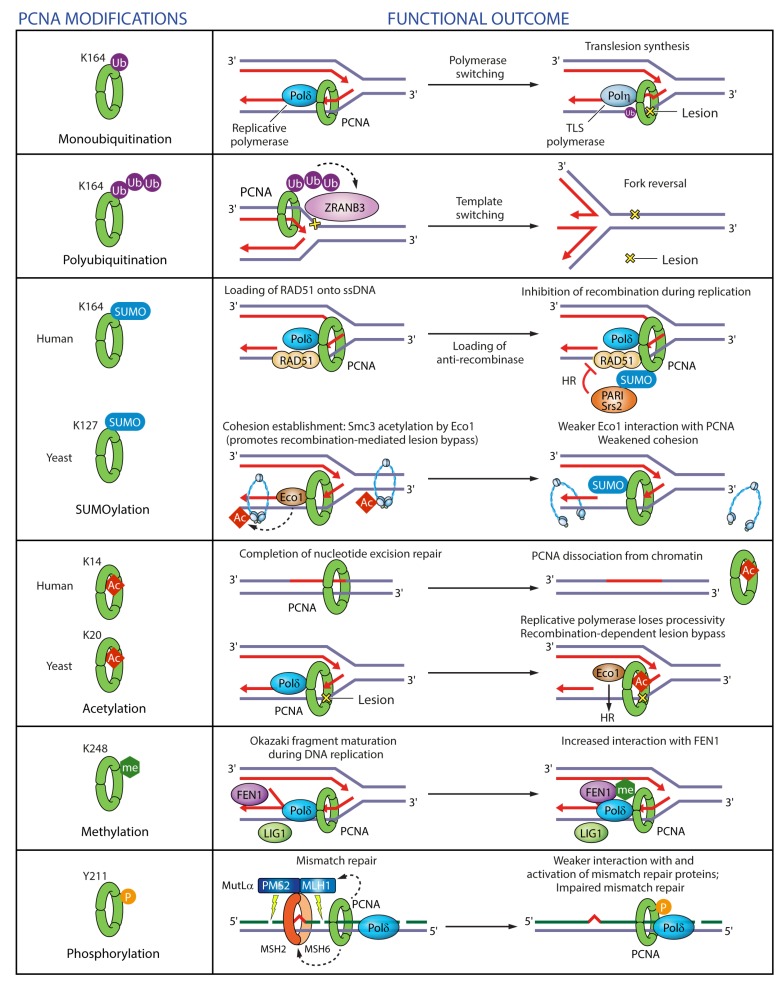
Regulation of PCNA functions in DNA replication and repair by post-translational modifications. PCNA monoubiquitinatation on K164 after DNA damage promotes ‘polymerase switching’, whereby stalled replicative polymerase is replaced with a specialized TLS polymerase. PCNA polyubiquitination on K164 after DNA damage triggers replication fork reversal through recruitment of ZRANB3 translocase/structure-specific endonuclease. PCNA SUMOylation on K164 recruits anti-recombinases PCNA-interacting protein (PARI) or Srs2 in human cells and yeast respectively to prevent recombination during replication. PCNA SUMOylation on K127 in yeast weakens the interaction of the acetyl transferase Eco1 with PCNA, which in turn impairs cohesion establishment. PCNA acetylation on K14 in human cells facilitates PCNA dissociation upon completion of DNA repair. PCNA acetylation on K20 in yeast reduces the processivity of Pol δ and promotes homologous recombination. PCNA methylation on K248 enhances its interaction with FEN1. PCNA phosphorylation on Y211 weakens its interaction with mismatch repair proteins.

Proliferating cell nuclear antigen (PCNA) is monoubiquitinated on K164 by RAD6-RAD18 in replicating S-phase cells as soon as polymerase stalling generates single-stranded DNA upon treatment with HU, MMS, crosslinking agents, or UV [60,63,109,110,111]. Monoubiquitinated K164 elicits ‘polymerase switching’, whereby stalled replicative polymerase is replaced with a specialized TLS polymerase, which contains a PIP-box and a UBD that mediates higher binding affinity for ubiquitinated PCNA [60] (Figure 8). Monoubiquitination of PCNA also occurs outside of S-phase following oxidative damage, to promote TLS polymerase recruitment to the lesions [112]. PCNA is also strongly ubiquitinated on K117 after UV damage [76], though the significance of this modification is unclear.

In addition to RAD6-RAD18, PCNA monoubiquitination may also be regulated by other ubiquitin ligases such as RNF8—the central ubiquitin ligase in signaling double-strand DNA breaks (DSBs) [113], and CRL4^Cdt2^, which monoubiquitinates PCNA in the absence of external damage together with RAD6-RAD18 [114]. Another regulatory mechanism that stabilizes PCNA monoubiquitination after UV damage is degradation of the USP1 deubiquitinase [115]. Conversely, PCNA monoubiquitination is removed by tethering USP10 deubiquitinase through ubiquitin-like ISG15 modification of PCNA on K164 and K168, leading to Pol η release and TLS termination [107]. PCNA K164 is NEDDylated at later time points after oxidative stress or UV damage and antagonizes K164 ubiquitination and recruitment of TLS polymerases [105,106,116]. The mono-ADP-ribosyltransferase PARP10 binds PCNA and stimulates PCNA monoubiquitination, although DNA damage-relevant substrates for mono-ADP-ribosylation are unknown [117]. Different non-modifying proteins were additionally reported to stimulate PCNA monoubiquitination following DNA damage, including SIVA1, Chk1, p21, p53, Claspin, Timeless, NBS1, ZBTB1, FANCD2, and PTIP [1,118,119].

Beyond monoubiquitination, DNA damage also induces further K63-linked polyubiquitination of K164 by ubiquitin-conjugating MMS2-UBC13 in complex with the yeast RAD5 or mammalian helicase-like transcription factor (HLTF) and SNF2 histone-linker PHD-finger RING-finger helicase (SHPRH) ubiquitin ligases [109,120,121]. Polyubiquitination is much more pronounced in yeast than in mammalian systems, though [119]. HLTF and SHPRH form mutually exclusive complexes that promote recruitment of specific TLS polymerases depending on the DNA damage type [122]. In their absence, however, PCNA can still be polyubiquitinated, pointing to the involvement of another ubiquitin ligase [123]. Polyubiquitination of PCNA triggers error-free template switch mechanisms (fork regression or strand invasion) [109,124] and recruits ZRANB3 translocase/structure-specific endonuclease [12,53] (Figure 8). ZRANB3 can induce replication fork slowing and reversal, disassemble D-loop recombination intermediates to prevent inappropriate recombination, and mediate DNA repair by acting as a structure-specific endonuclease [12,13,53].

As opposed to ubiquitination, SUMOylation of PCNA on K164 during S-phase suppresses homologous recombination by recruiting PCNA-interacting protein (PARI) as an anti-recombinase in mammalian cells [125] or the Srs2 anti-recombinase in yeast [126,127] (Figure 8). In addition, PCNA SUMOylation prevents DSB formation under replication stress conditions induced by MMS or cisplatin, but not UV [98]. In yeast, SUMOylation of PCNA on K127 antagonizes PCNA-dependent Eco1 function and thus abrogates sister chromatid cohesion [128] (Figure 8).

Acetylation is another PTM relevant for PCNA regulation. Acetylation of lysine residues at the inner surface of the PCNA ring negatively regulates PCNA interactions with other proteins by modulating PCNA-DNA binding rather than PCNA-protein interactions [5]. In S-phase yeast cells exposed to the DNA-damaging agent MMS, PCNA is acetylated on K20 by Eco1 at the inner surface of the PCNA ring [129]. Lysine acetylation at the PCNA-DNA interface loosens PCNA-DNA contact resulting in a change in the DNA tilt that impairs Pol δ assembly [4,5]. Consequently, K20ac reduces the processivity of the replicative polymerase Pol δ and promotes HR-mediated repair of DNA lesions in replicating cells [129] (Figure 8). In human cells, acetylation on K14 at the inner surface by CBP and p300 acetyl transferases was shown to negatively regulate PCNA interaction with MTH2 in response to UV damage, resulting in PCNA susceptibility to proteosomal degradation and dissociation from DNA damage sites [104,130] (Figure 8). Removal of PCNA from chromatin upon completion of NER is necessary to prevent genomic instability.

The only methylation site on PCNA identified to date, K248, is deposited by SETD8, and was shown to stabilize PCNA protein levels and enhance the interaction between PCNA and FEN1 [108] (Figure 8). The functional relevance of other modifications on this residue is currently unclear.

Mass spectrometry analyses identified 13 phosphorylation sites on PCNA, of which Y211 and S261 seem to be most prevalent [131,132,133,134,135,136,137,138] (Figure 7 and Supplementary Table S1). Phosphorylation of PCNA on Y211 by the epidermal growth factor receptor (EGFR) enhances the stability of chromatin-bound PCNA but also weakens its interaction with mismatch repair proteins and thus suppresses the mismatch repair pathway [134,135] (Figure 8). Furthermore, a crosstalk between phosphorylation and ubiquitination seems to be important for replication stress response in light of the recent findings that phosphorylation of Y60, Y133 and Y250 by IGF-1R promotes PCNA ubiquitination and replication fork restart after UV or MMS exposure [131].

PCNA is heavily ADP-ribosylated on aspartate and glutamate residues in response to oxidative stress induced by H_2_O_2_, and is the only modification concentrated in the IDCL region of PCNA [139] (Figure 7 and Supplementary Table S1). Considering that ADP-ribose units can polymerize into long chains on poly(ADP-ribose) (PAR), this modification can potentially perturb the binding of PIP-box or APIM-containing proteins to IDCL through an increase in negative charge from PAR pyrophosphates [140].

## 5. Post-Translational Modifications of PCNA-Interacting Proteins

It is clear that PTMs on PCNA have important regulatory functions. This implies that PCNA-interacting proteins may also be regulated by PTMs [80]. Interaction interfaces with PCNA, such as PIP-boxes and UBDs, often reside in structurally disordered regions, which are highly amenable to PTMs (Figure 7). Structural flexibility of these regions allows multivalent binding of the PIP-box and UBD to the IDCL loop and the PCNA ubiquitination site respectively [49,165] (Figure 5A).

The best insight into the regulation of PCNA-binding proteins by PTMs comes from TLS polymerases. Y-family TLS polymerases are able to bypass DNA lesions due to their spacious active site that can accommodate non-canonical Watson-Crick base pairing caused by DNA lesions [166]. Pol η can accurately bypass thymine-thymine cyclobutane pyrimidine dimers (CPDs) caused by UV as well as cisplatin-induced crosslinks [167]. A Pol η mutation has been associated with a variant form of the genetic disease Xeroderma pigmentosum (XP-V) characterized by sun sensitivity and predisposition to skin cancer [168,169]. XP-V cells exhibit defects in repair replication and elevated mutagenesis after UV exposure due to inefficient and inaccurate bypass of UV lesions without the functional Pol η [170,171]. In addition to TLS, Pol η has been implicated in the replication of common fragile sites (CFSs) [172,173]. Pol ι can also bypass UV lesions but with reduced fidelity [174]. It shows specificity for the misincorporation of dG over the correct dA opposite T/U [175]. Pol κ specializes in the bypass of bulky adducts generated by polycyclic aromatic hydrocarbons and is highly efficient in the extension of mismatched termini [176,177]. REV1 acts as a ‘polymerase bridge’ to facilitate switching from the insertion to the extension step of lesion bypass [73]. Given that TLS polymerases have different substrate specificities and exhibit low fidelity on undamaged templates, their localization and activity need to be tightly regulated.

**Figure 9:**
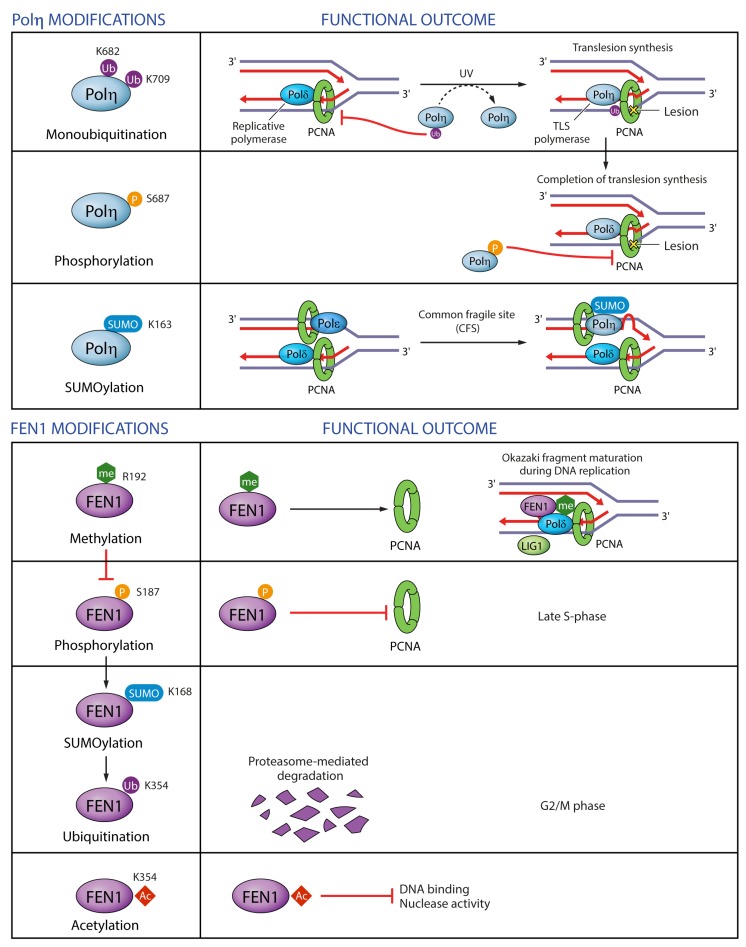
Regulation of PCNA-interacting proteins by post-translational modifications. Examples are given for two PCNA-interacting proteins: Pol η and FEN1. Monoubiquitination of Pol η suppresses monoubiquitinated PCNA and Pol η-mediated translesion synthesis. Pol η phosphorylation weakens its binding to PCNA and may promote its dissociation from replication forks upon completion of translesion synthesis. Pol η SUMOylation promotes Pol η recruitment to difficult-to-replicate common fragile sites. FEN1 methylation promotes its interaction with PCNA during Okazaki fragment maturation. FEN1 methylation antagonizes its phosphorylation, which abrogates its binding to PCNA. FEN1 phosphorylation promotes its SUMOylation and ubiquitination leading to FEN1 proteasome-mediated degradation in G2/M phase of the cell cycle. FEN1 acetylation negatively regulates its binding to DNA and its nuclease activity.

Monoubiquitination of PCNA promotes PCNA interaction with TLS polymerases and stimulates their activity and processivity, while Pol η and κ in turn promote PCNA monoubiquitination [58,66,178]. Conversely, monoubiquitination of Pol η in the vicinity of the PIP-box (within NLS) abolishes its binding to PCNA in undamaged cells presumably due to intramolecular interaction between ubiquitin and UBD [71]. Importantly, UV damage elicits Pol η deubiquitination on K682 and K709, enabling recruitment to stalled replication forks [71,76] (Figure 9). Unlike ubiquitination, SUMOylation on K163 recruits Pol η to replication forks and reinforces the role of Pol η in the replication of difficult-to-replicate CFSs, but is not required for TLS of UV-induced lesions [65] (Figure 9). This is a nice example of how two different PTMs, ubiqutination within the NLS and SUMOylation within the catalytic region, can differentially regulate two separate functions of a TLS polymerase: its bypass of UV lesions on the one hand, and replication of difficult loci on the other. Considering that Pol η has 48 lysine residues, of which 19 can be ubiquitinated and 20 SUMOylated, with 10 of them overlapping [69,71,76,81,82,83,84,89,90,91,143,160] (Figure 7 and Supplementary Table S1), it seems likely that other ubiquitinated or SUMOylated residues are also implicated in the regulation of Pol η interactions with PCNA or other proteins, or in the regulation of Pol η catalytic activity.

Pol η NLS is not only ubiquitinated, but also phosphorylated on S687 after UV [160]. Like ubiquitination, phosphorylation diminishes Pol η binding to PCNA, and was proposed to mediate removal of Pol η upon completion of TLS [160] (Figure 9). In general, phosphorylation of Pol η in response to DNA damage serves a positive regulatory function by promoting Pol η foci formation, TLS efficiency and cell survival [160,179,180].

Pol ι is also known to be extensively monoubiquitinated (24/54 lysines) and SUMOylated (9 lysines; 8 overlapping with ubiquitin), but the function of monoubiquitination or SUMOylation in this protein remains underexplored [69,80,81,82,83,84,85,86,89,90,91,99,143] (Figure 7 and Supplementary Table S1). Mass spectrometry analysis of UV-induced PTM changes revealed a decrease in Pol ι ubiquitination on K471 after UV [143]. Monoubiquitination of Pol η or Pol ι was shown to enhance their mutual binding, which facilitates Pol ι localization within replication foci [181], and two ubiquitinated lysines in Pol ι are within the PIP-box [83,84]. Lysines in proximity to UBM1 of Pol ι can be not only ubiquitinated or SUMOylated but also methylated (K551 and K555), suggesting that this region is actively regulated by PTM switching [154].

Of the three TLS polymerases, Pol κ is most heavily ubiquitinated and SUMOylated with 34 and 47 modified lysines out of 84 respectively, also within the PIP-box and UBZ motifs [76,82,83,84,89,90,97,101,143] (Figure 7 and Supplementary Table S1). UV damage induces deubiquitination of K443, which resides within the catalytic region and may regulate Pol κ activity [76].

Another PCNA-interacting protein implicated in DNA replication and repair, PARG, is extensively modified by PTMs in the regulatory region, which may regulate its interaction with PCNA [27]. PARG is an essential enzyme in eukaryotes required for removal of poly(ADP-ribose) (PAR). PARG is necessary for replication and recovery from prolonged replication stress [27,182,183,184,185]. PARG-deficient cells proliferate more slowly and accumulate abnormal DNA replication intermediates, while PARG inhibition stalls replication forks and decreases the efficiency of homologous recombination (HR) [182,183,184,185]. PARG is recruited to DNA damage sites in a PCNA-dependent fashion [27,141]. The N-terminal regulatory region of PARG is unstructured and bears many acetylation and phosphorylation sites [27,82,87,88,93,100,132,136,137,138,139,143,148,149,151,159] (Figure 7 and Supplementary Table S1). One of the acetylation sites, K409, resides within the PARG PIP-box and may negatively regulate PARG interaction with PCNA, thus promoting PARG dissociation from DNA damage sites as in the case of PCNA acetylation [27,130].

The regulation of the PCNA-interacting protein FEN1 by PTMs has been well studied, with several insights into the regulation of its interactions with PCNA (Figure 9). FEN1 interaction with PCNA is dually regulated through phosphorylation and methylation of residues outside of its PIP-box. Methylation of R192 promotes FEN1 interaction with PCNA, replication foci formation, Okazaki fragment maturation, normal cell cycle progression but also BER following oxidative damage [145]. R192 methylation antagonizes phosphorylation of FEN1 on S187, which is deposited in late S-phase by Cdk1-Cyclin A and abrogates PCNA binding [142,145]. FEN1 phosphorylation on S187 promotes its SUMOylation on K168, which in turn induces its ubiquitination on K354 and the resulting proteasome-mediated degradation in G2/M phase of the cell cycle [146]. Cells expressing degradation-resistant FEN1 K168R and K354R mutants exhibit cell cycle delays and chromosomal missegregation [146]. K354 together with K375, K377 and K380 is also acetylated by p300 after UV exposure and negatively regulates the binding of FEN1 to DNA and its nuclease activity, without affecting its interaction with PCNA [161]. A dynamic interplay between many different PTMs thus seems to be critical for the regulation of FEN1 functions in DNA replication and repair.

Phosphorylation also negatively modulates interactions between other PIP-box-containing proteins and PCNA, such as the CDK inhibitor p21 and the p68 subunit of Pol δ [162,163]. While in the case of FEN1 the interaction with PCNA is regulated by phosphorylation of a serine residue outside of the PIP-box, presumably through a conformational change that masks the PIP-box, in the case of p21 and Pol δ the regulatory phosphorylation marks occur within the PIP-box itself [142,162,163]. Phosphorylation within the PIP-box disrupts hydrophobic interactions and thereby weakens the binding of p21 and Pol δ to PCNA [162,163]. Phosphorylation of p21 may support unperturbed replication by allowing the loading of replication factors, while its dephosphorylation may halt replication in response to DNA damage [162]. Phosphorylation of the Pol δ PIP-box and FEN1 may enable switching between Pol δ, FEN1 and DNA ligase I during Okazaki fragment maturation, or between Pol δ and TLS polymerases [163]. The functional relevance of these phosphorylations, however, remains to be addressed.

## 6. Perspective

The scaffold role of PCNA in DNA replication is essential for normal cellular proliferation, uncontrolled proliferation being one of the hallmarks of cancer. In DNA replication, PCNA orchestrates the recruitment of enzymes involved in lagging strand synthesis and Okazaki fragment maturation, whereby phosphorylation of DNA polymerase δ, flap endonuclease FEN1 and DNA ligase I likely ensures their sequential recruitment to PCNA [163]. Moreover, the role of PCNA in DNA repair is critical for the regulation of the access of DNA repair proteins to DNA damage sites such as error-free fork reversal proteins or error-prone TLS polymerases. On the one hand, impaired ubiquitination of PCNA or inability of TLS polymerases to be recruited to ubiquitinated PCNA through UBD deletion sensitizes the cells to DNA damage [45,61,71,72]. On the other hand, uncontrolled access of TLS polymerases to replication forks may enhance mutagenesis thus contributing to oncogenic transformation [186]. Regulation of PCNA-mediated interactions is thus critical for normal cellular function, underlying the importance of post-translational modifications of both PCNA and its interactors.

PCNA is an attractive target for cancer therapy and PCNA levels are increased in some cancer cells [187,188]. Significant progress has been made in the development of specific PCNA inhibitors. A peptide spanning the IDCL (126–133aa) was shown to reduce PCNA association with chromatin and exert cytotoxic effect on breast cancer, lymphoma, and pancreatic cancer cell lines [189]. Alternative IDCL targeting through a non-peptide small molecule inhibitor was shown to inhibit binding of p21 and Pol δ to PCNA and to interfere with DNA replication [190]. However, targeting IDCL as a common interaction site for many different PIP-box-containing proteins may cause general cytotoxicity and severe side effects often associated with cancer treatment. Targeting PTMs on PCNA or the regulatory domain of PCNA-interacting proteins may instead prove to be more specific. Inhibition of PCNA phosphorylation on Y211 using a synthetic peptide was shown to inhibit proliferation of prostate cancer cells and reduce tumor growth in xenograft prostate tumors [191]. Identification of specific residues that are regulated by PTMs and that modulate the binding to PCNA, would allow the design of small molecule inhibitors that target these specific residues, and would specifically interfere with a particular function of PCNA, such as TLS. Such inhibitors when combined with chemotherapy to induce DNA damage would specifically interfere with the function of PCNA-binding proteins in facilitating recovery of highly proliferating cancer cells from increased DNA damage burden. Further efforts in uncovering the role of a myriad sites of various PTMs on PCNA and PCNA interactors will shed light on how PCNA can coordinate the dynamic exchange of its many partners in DNA replication and repair.

## Figures and Tables

**Table 1:** Sequence alignment of PIP-boxes for which binding affinities and/or crystal structures with PCNA are available. Binding affinities are represented as dissociation constant (K_d_). The values are not fully comparable due to variable methods and conditions in which they were obtained, as well as different peptide length/sequence. When available, more than one binding affinity from different publications is indicated. The following binding assays were employed: isothermal titration calorimetry (ITC) [10,19,22,27,33,34,35,36,37]; surface plasmon resonance (SPR) [21]; fluorescence polarization assay (FPA) [20].

Protein	PIP-Box Peptide	K_d_ (PCNA)	PDB
p21	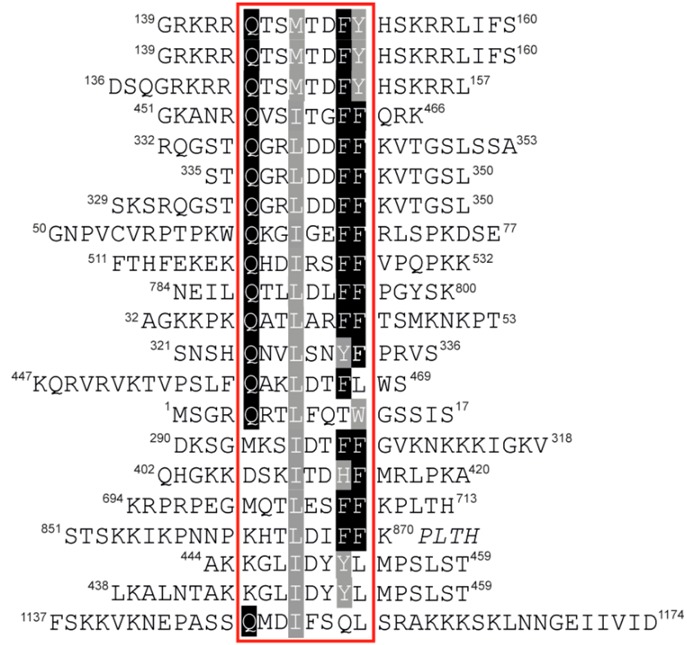	0.08 μM [22] 0.56 μM [33] 0.28 μM [19]	1AXC [31]
Pol δ p68		15.6 μM [22]	1U76 [22]
FEN1		59.9 μM [22] 9.7 μM [33] 17.3 μM [19]	1U7B [22] 1UL1 [38]
PAF15		5.6 μM [34]	4D2G [34]
ZRANB3		4.8 μM [19]	5MLO [19]
UHRF2		25.7 μM [35]	5YCO [35]
Cdc9			2OD8 [39]
DVC1		15.6 μM [36]	5IY4 [36]
TRAIP		30.7 μM [37]	4ZTD [37]
FANCM		13.0 μM [10]	
RNASEH2B		35.0 μM [33]	3P87 [40]
PARG		3.3 μM [27]	5MAV [27]
Pol η		0.4 μM [21]	2ZVK [21]
Pol κ		4.9 μM [21]	2ZVL [21]
Pol ι		0.4 μM [21] 5.5 μM [19]	2ZVM [21]
Srs2		0.6 μM [20]	3V62 [20]

## Supplementary Materials

The following are available online at http://www.mdpi.com/2073-4425/9/8/416/s1, Supplementary Table S1: Post-translational modifications of PCNA and PCNA-interacting proteins. Pubmed IDs of resource publications are provided for each modified residue.
